# Supplementary material for: DNA Interaction with a Polyelectrolyte Monolayer at Solution—Air Interface
Source: Polymers (Basel). 2021 Aug 22;13(16):2820. doi: 10.3390/polym13162820 (PMC8400178; doi:10.3390/polym13162820)
Supplement: Supplementary file 1 [file polymers-13-02820-s001.zip › polymers-1337851-supplementary.pdf]

DNA Interaction with a Polyelectolyte Monolayer at Solution – Air Interface

N.S. Chirkov, R.A. Campbell, A.V. Michailov, P.S. Vlasov, B.A. Noskov*

St.Petersburg State University

Supplementary material

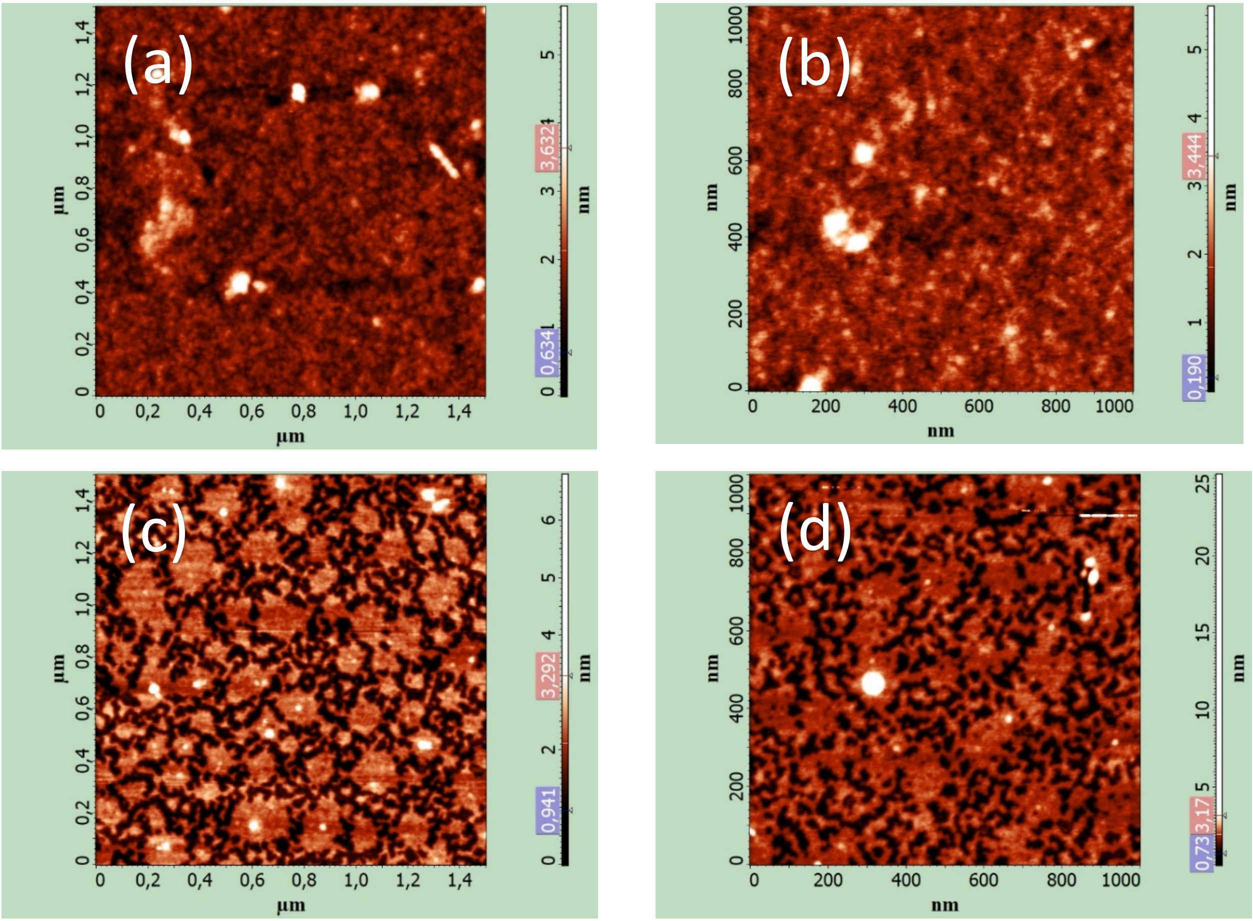

Figure S1. AFM images of spread PDAHMAC films transferred from a liquid surface onto a surface of mica at different surface pressures: 12 (a, b) and 24 (c, d) mN/m.

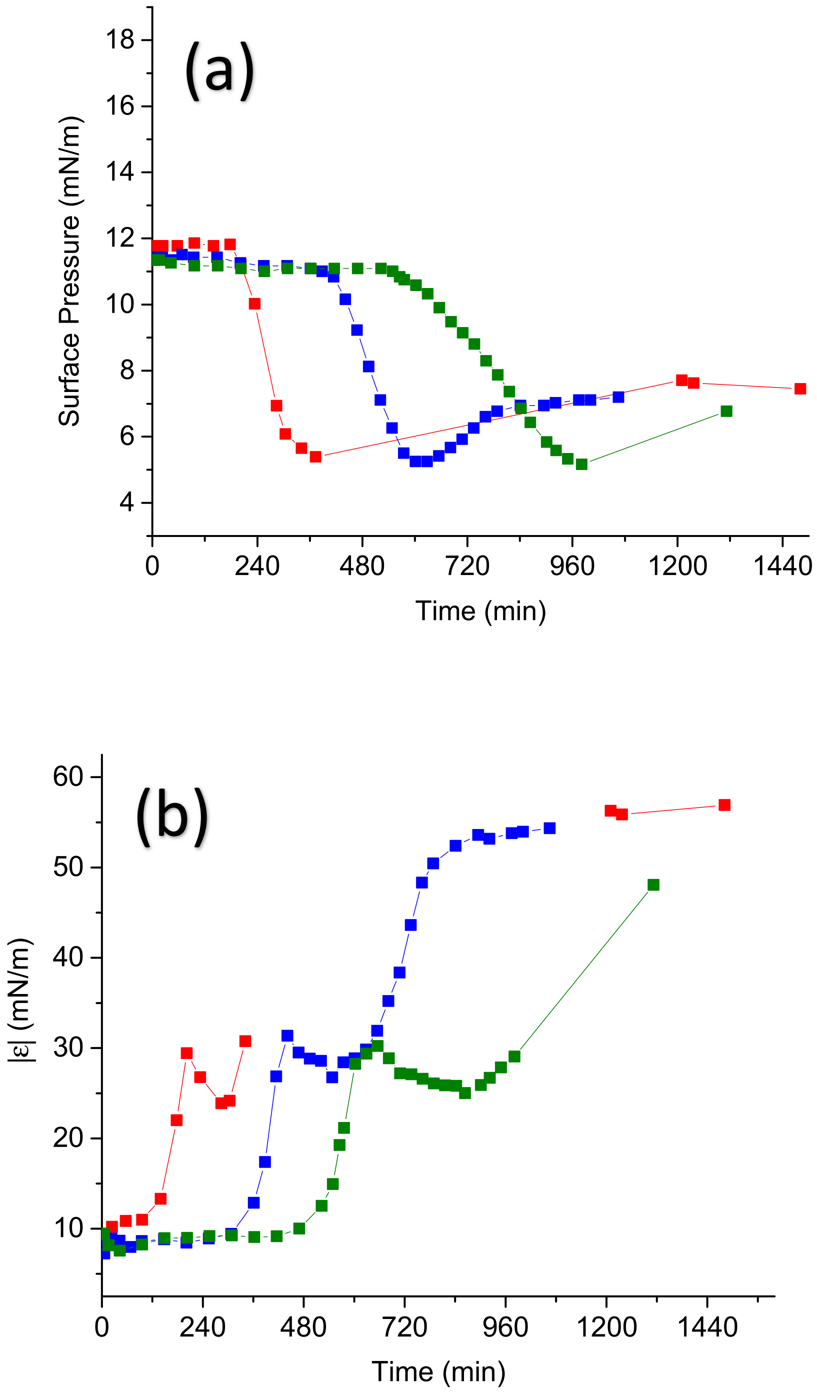

Figure S2. Kinetic dependences of the surface pressure (a) and the dynamic elasticity (b) of PDAHMAC layers after a DNA injection into the subphase. The total DNA concentration is 28 μM and the initial surface pressure is 11 mN/m. The three sets of experimental data correspond to different independent measurements. Lines are guides for an eye.

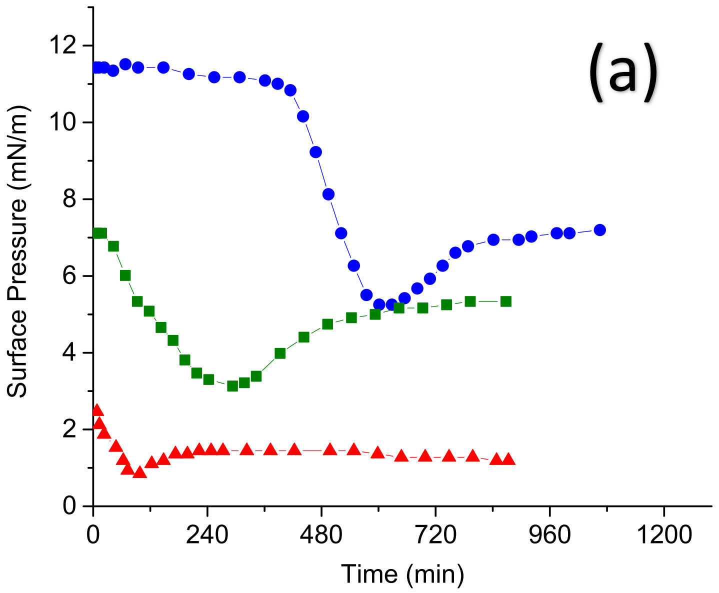

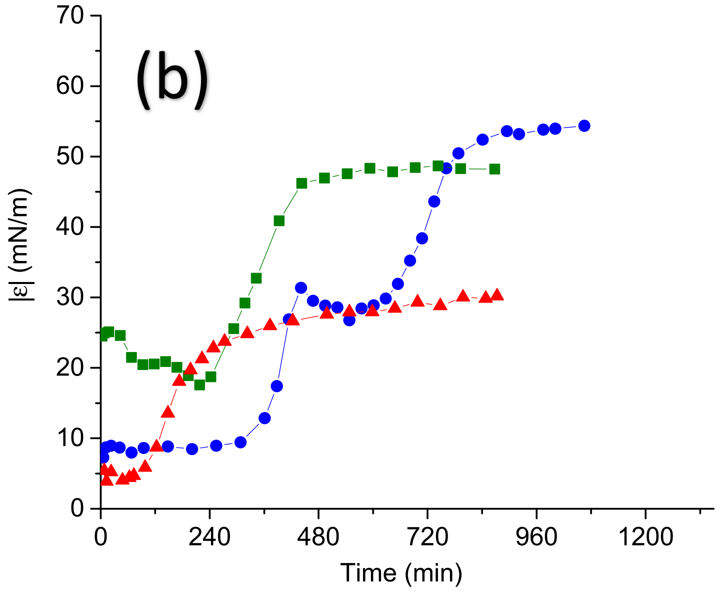

Figure S3. Kinetic dependences of the surface pressure (a) and the dynamic elasticity (b) of PDAHMAC layer after a DNA injection into the subphase at initial surface pressures: 11 (blue circles), 7 (green squares) and 2 (red triangles) mN/m. The DNA concentration is 28 μM. Lines are guide for an eye.

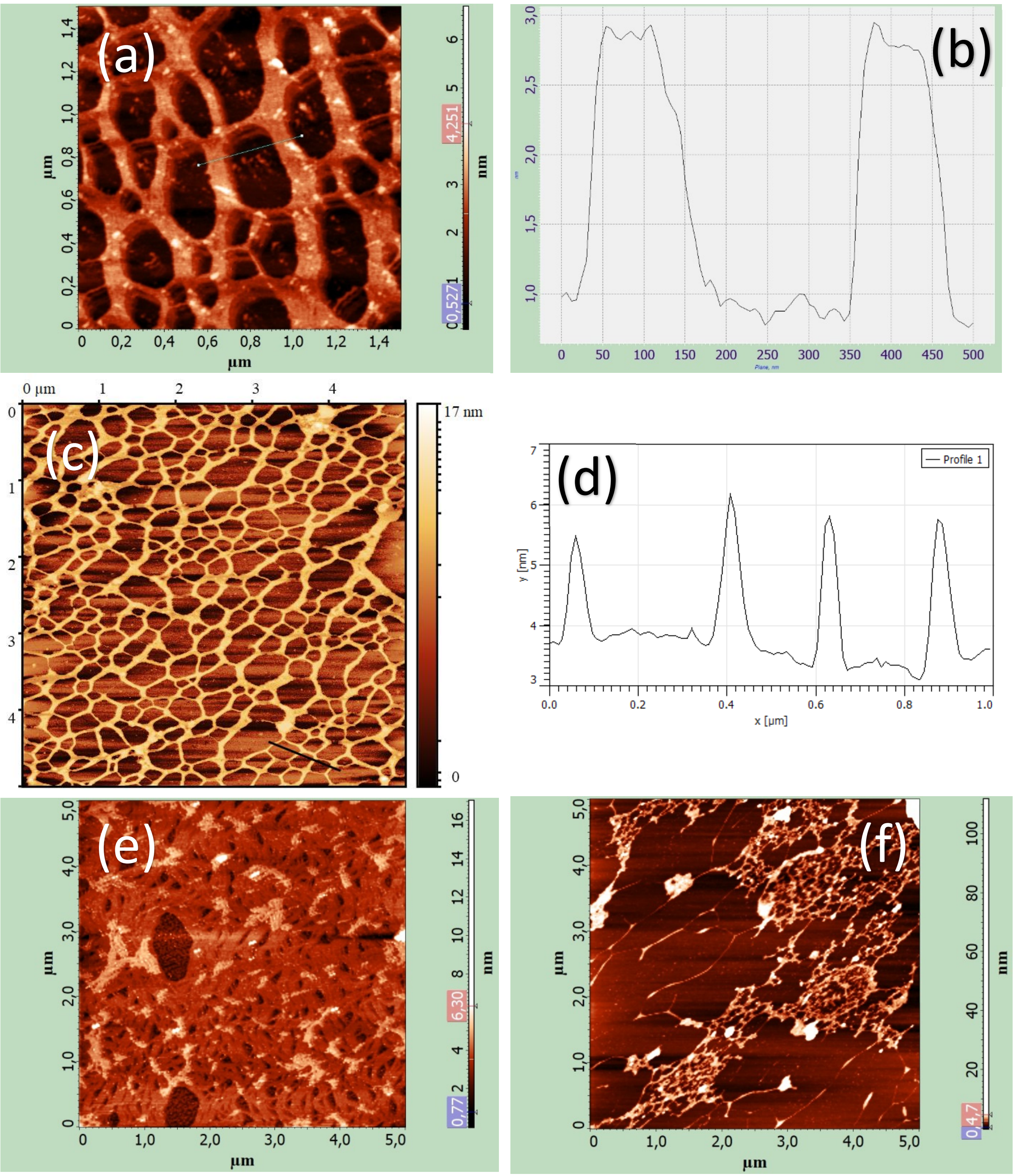

Figure S4. AFM images of spread DNA/PDAHMAC layers transferred onto the mica surface after a DNA injection at stationary values of the dynamic surface properties and initial surface pressures of 11 (a,e,f) and 7 mN/m (c). Graphs (b) and (d) present the cross-sections of the aggregates in figures (a) and (c), respectively.
